# The Role of Structural Extracellular Matrix Proteins in Urothelial Bladder Cancer (Review)

**DOI:** 10.4137/bmi.s294

**Published:** 2007-11-05

**Authors:** Andrea Brunner, Alexandar Tzankov

**Affiliations:** 1 Institute of Pathology, Medical University of Innsbruck, Innsbruck, Austria; 2 Institute of Pathology, University of Basel, Basel, Switzerland

**Keywords:** bladder cancer, extracellular matrix, laminin, fibronectin, tenascin, thrombospondin 1

## Abstract

The extracellular matrix (ECM) plays a key role in the modulation of cancer cell invasion. In urothelial carcinoma of the bladder (UC) the role of ECM proteins has been widely studied. The mechanisms, which are involved in the development of invasion, progression and generalization, are complex, depending on the interaction of ECM proteins with each other as well as with cancer cells. The following review will focus on the pathogenetic role and prognostic value of structural proteins, such as laminins, collagens, fibronectin (FN), tenascin (Tn-C) and thrombospondin 1 (TSP1) in UC. In addition, the role of integrins mediating the interaction of ECM molecules and cancer cells will be addressed, since integrin-mediated FN, Tn-C and TSP1 interactions seem to play an important role during tumor cell invasion and angiogenesis.

## Introduction

Originally the extracellular matrix (ECM) was seen as “glue” between other important elements, but proved to be a dynamic structure, playing a key role in fetal development, tissue repair and angiogenesis as well as in modulation of cancer cell invasion (Bosman and Stamenkovic, 2003). The ECM constitutes of structural proteins, such as collagens, elastin, laminins and glycoproteins such as fibronectin (FN), vitronectin and tenascin (Tn-C) and a variety of other proteins including proteolytic enzymes such as matrix metalloproteinases and their inhibitors as well as proteoglycans (Bosman and Stamenkovic, 2003). In the progression to invasive cancer, complex interactions are necessary to pass the basal membrane, invade the surrounding tissue and vessel walls. In all these processes, the ECM is critically involved (Bosman and Stamenkovic, 2003).

In the normal urinary bladder, structural proteins of the wall are necessary to maintain the integrity of the impermeable bladder surface (Wilson et al. 1996). Disturbance of these proteins has been suggested to be involved in inflammatory conditions (Deen et al. 1994). In urothelial carcinoma of the bladder (UC), the role of ECM proteins has been widely studied to assess invasive behavior. Gene expression in bladder cancer cell lines has been shown to depend on the matrix used for cultivation (Deen et al. 1994; Ioachim et al. 2005; Dozmorov et al. 2006). The following review will focus on the role of structural proteins in UC, which have been suggested to be of prognostic value (Table 1).

## Extracellular Matrix in Urothelial Bladder Cancer

### Laminin

Laminin is one of the most important components of the basal membrane. First described 20 years ago, 15 distinct laminin isoforms have been isolated so far (Bosman and Stamenkovic, 2003). The molecule is built of three disulfide-linked chains, including five α, three β and three γ isoforms, with a characteristic cross shape (Bosman and Stamenkovic, 2003). Laminin is produced by nearly all epithelial-, smooth muscle-, cardiac muscle-, nerve- and endothelial cells (Bosman and Stamenkovic, 2003). Functionally, depending on the isoforms expressed, laminins are involved in cell proliferation, adhesion and migration (Engbring et al. 2003). Their effects are mediated through binding to integrins and their most important function seems to be the interaction between epithelial cells and the ECM (Syrigos et al. 1999; Bosman and Stamenkovic, 2003). The role of laminins in cancer has been excessively studied and it has been shown that aberrant synthesis, chain composition and proteolytic modification are important for the interaction between malignant cells and ECM (Pattarroyo et al. 2002).

In UC of the bladder, the distribution of laminins has been studied in order to assess infiltrative behavior, to detect early invasion and to evaluate the presence of tumor-derived basal membranes (Conn et al. 1987; Schapers et al. 1990; Abou Fahra et al. 1993). Several studies focused on the usefulness of immunohistochemical staining for basal membrane components and their prognostic significance (Conn et al. 1987; Schapers et al. 1990; Abou Fahra et al. 1993). Schapers et al. found a significant association between the presence of intact basal membranes and stage (Schapers et al. 1990). In cases where stage could not be determined on hematoxylin and eosin stained slides, staining for basal membrane components proved to be helpful in determining the correct stage (Schapers et al. 1990). Recurrence-free survival was not influenced by these findings, though a tendency towards a higher risk of progression and worse overall survival for patients, whose tumors showed disrupted basal membranes, was reported (Schapers et al. 1990). Interestingly, some clearly invasive tumors in this study showed an intact basal membrane (Schapers et al. 1990). It was therefore suggested, that a positive relation between breakdown and deposition of basal membrane components probably reflects an intact host response to the neoplasm (Conn et al. 1987; Schapers et al. 1990). In fact, the basal membrane is not a static, but a dynamic structure, characterized by constant deposition and degradation of its components (Schapers et al. 1990). Alampi et al. observed a worse overall survival for patients with loss or fragmentation of the basal membranes compared to those with intact basal membranes (Alampi et al. 1989). Abou Fahra et al. found a higher recurrence rate and shorter recurrence-free survival for patients with disrupted basal membranes; those with lost stainable basal membranes around vessels more commonly developed metastatic disease (Abou Fahra et al. 1993). On the other hand, Ioachim et al. evaluated the distribution of ECM components in bladder cancer and reported that the expression of laminin did not contribute to recurrence and progression in UC (Ioachmin et al. 2005).

Laminins are a heterogeneous group of proteins with different isoforms, which mediate a diversity of functions (Hindermann et al. 2003). One isoform, involved in the formation of hemidesmosomes in basal epithelial cells that was identified to be of importance for cellular adhesion and migration, was laminin-5 (Hindermann et al. 2003). The function of laminin-5 is known to depend on proteolytic processing through plasmin and matrix metalloproteinases (Bosman and Stamenkovic, 2003). In fact, the γ2 chain, unique to laminin-5, proved to be of importance for the migration and cell adhesion of malignant tumors (Gianelli et al. 2001; Hindermann et al. 2003). Several studies assessed the value of the γ2 chain in different tumor types, with both reported losses as well as increased expression (Sordat et al. 1998; Kosmehl et al. 1999; Ono et al. 1999; Skyldberg et al. 1999; Henning et al. 1999; Gianelli et al. 2001; Hao et al. 1996). The γ2 chain was found to be expressed in ECM also outside the basal membrane and to be retained in tumor cells of superficial UC, while in invasive tumors, loss of basal membrane staining was evident (Hindermann et al. 2003). These findings were associated with a worse overall survival, but independent prognostic significance was not reached. This altered distribution suggests a failure to form anchoring filaments and hemidesmosomes allowing carcinoma cell invasion (Hindermann et al. 2003). Furthermore, in patients treated with transurethral resection (TUR), but not cystectomy, the deposition of γ2 chain of laminin-5 proved to be an independent prognostic factor indicating a higher risk of recurrence (Kiyoshima et al. 2005). The expression of γ2 chain was not influenced by the expression of epidermal growth factor receptor (EGFR) and human epidermal growth receptor 2 (HER2/neu) as well as cyclooxygenase 2, which have also been found to be associated with more aggressive UC behavior and probably directly interact with the γ2 chain (Kiyoshima et al. 2005). Further, particularly prospective, studies should provide additional information on the interactions and functions of the γ2 chain in UC in order to establish its diagnostic, prognostic and predictive significance.

### Collagens

Collagens play an important role as a scaffold in maintaining tissue structure (Bosman and Stamenkovic, 2003). Collagens are either organized as fibrils in tissues that are exposed to shear, tensile or pressure forces including tendons, bone cartilage and skin, or are able to form networks, such as collagen IV, which is an important component of the basal membrane (Bosman and Stamenkovic, 2003). Collagens are usually synthesized by mesenchymal cells, such as fibroblasts and myofibroblasts, but collagen IV is also produced by adjacent epithelial cells (Bosman and Stamenkovic, 2003). In hollow organs, the most important interstitial collagens include collagens III and I (Bosman and Stamenkovic, 2003). In the normal bladder wall, these two types are mainly expressed in the lamina propria and around smooth muscle bundles and nerves (Wilson et al. 1996). Interstitial collagens have been suggested to be involved in rat bladder cancer cell lines single cell infiltration (Tucker et al. 1990). The role of collagens in UC has not been extensively studied, except for collagens IV and VII, known to be basal membrane components; collagen VII playing a role in the formation of hemidesmosomes with the basal cells of the urothelium (Daher et al. 1987; Schapers et al. 1990; Liebert et al. 1994). Similar to laminin, collagen IV staining was used to assess the presence of early invasion (Daher et al. 1987; Schapers et al. 1990). The loss of collagen IV expression in the basal membrane was reported to be associated with a worse overall survival and a tendency towards progression, but no influence on recurrence-free survival could be detected (Schapers et al. 1990). Daher et al. investigated collagen IV staining pattern in a group of invasive cancers and found that widely absent or fragmented staining in more than 5% of a tumors predicted a worse short term survival (Daher et al. 1987). Özer et al. focused on high grade pT1 tumors and found that collagen IV did not play a role in predicting behavior (Özer et al. 1999). It is difficult to assess if collagen IV can be helpful in evaluating the correct tumor stage, since it may be absent in urothelium with substantial superficial inflammation, a condition that can also accompany carcinoma in situ and papillary neoplasms (Deen et al. 1994). Collagen VII has been studied in UC together with the expression of integrin α6β4, which is, in normal urothelium, co-localized with collagen VII (Liebert et al. 1994). Loss of this co-localization has been found and the degree of de-arrangement increased in invasive cancers, suggesting involvement during invasion (Liebert et al. 1994). Since its particular sensitivity to inflammatory conditions, alteration of collagen stainability could be of little practical importance concerning diagnosis and prognosis of UC.

### Fibronectin

Fibronectin (FN) is a non-collagenous glycoprotein with a distinct tissue distribution, predominantly localized in mesenchymal tissues (Ioachim et al. 2005). Similar to laminin, FN plays a role in cell adhesion, proliferation and migration (Ioachim et al. 2005). The distribution of FN in the normal bladder wall and in UC has been described by few authors (Deen and Ball, 1994; Wilson et al. 1996). The normal bladder wall revealed no consistent staining in the urothelium, though some superficial urothelial cells were positive (Wilson et al. 1996). Staining in the lamina propria was present and enhanced just beneath the basal membrane as well as surrounding individual muscle fibers and in vessel walls (Wilson et al. 1994). FN staining in UC appeared to be intense around invasive tumors cells nests, while in non-invasive tumors staining seemed to be more heterogeneous and mostly enhanced in the lamina propria, but only faintly present in the tumor papillary cores (Deen et al. 1994; Ioachim et al. 2005). Data on the prognostic value of FN in UC are scarce. Ioachim et al. found that expression of FN was associated with tumor stage, proliferation index, laminin, collagen IV, tenascin C (Tn-C), and microvessel density, suggesting that FN is associated with proliferation, invasion and angiogenesis (Ioachim et al. 2005). In fact, it is well known that FN interacts with Tn-C, which modulates FN-dependent adhesions, thus probably contributing to invasive behavior (Huang et al. 2001). FN staining in the study of Ioachim et al. was not associated with progression or recurrence (Ioachim et al. 2005). The putative value of FN was otherwise only assessed in urine or blood of UC patients as well as in tissue homogenates of tumors. Kirkali et al. found an increase of FN in tissue homogenates of patients with UC, when compared to healthy controls (Kirkali et al. 2001). Some studies focused on FN levels in urine (Malmstrom et al. 1993; Menendez et al. 2005). In the presence of malignant tumors, the components of the ECM are degraded by proteases during invasion (Menendez et al. 2005). Antibodies against specific cell binding domains of FN allow the detection of fragments in urine (Katayama et al. 1991, 1993). It has been reported that cancer patients have increased levels of urinary FN compared to healthy persons (Katayama et al. 1991, 1993). For this purpose, an automatic assay called bladder tumor fibronectin (BTF) was developed (Katayama et al. 1991; Menendez et al. 2005). Menendez et al. performed a prospective study in 123 patients to evaluate the diagnostic accuracy of BTF (Menendez et al. 2005). They reported that levels of urinary FN were substantially elevated in patients with UC compared to healthy controls, but the test was limited by lack of sensitivity in low grade tumors as well as lack of specificity in patients with infections of the lower urinary tract, benign or malignant prostatic disease, urinary stones or after endovesical chemotherapy (Menendez et al. 2005). Malmstrom et al. found decreased urinary FN after transurethral tumor resection as well as in patients with response after Bacillus Calmette-Guérin (BCG)-treatment suggesting that urinary FN might be helpful in selecting patients for further BCG-therapy (Malmstrom et al. 1993). On the other hand, Danisman et al. reported that urinary FN is of no additional value in patients treated with BCG (Danisman et al. 2000).

Due to low sensitivity, determination of FN in plasma of UC patients did not prove to be helpful (Hegele et al. 2003). Indeed, the main percentage of FN in the blood is synthesized by hepatocytes, but one distinct form the so called cellular fibronectin (cFN) is derived from other cells and has a specific domain created by alternative mRNA splicing by which it can be detected in plasma (Hegele et al. 2003). Hegele et al. studied cFN in urine and blood from 20 UC patients and 20 controls using a highly sensitive time resolved fluorescence immunoassay (TRIFA) (Hegele et al. 2003). cFN levels in plasma were significantly higher in cancer patients compared to controls and differences were also detected between pTa/pT1 and pT2–4 tumors (Hegele et al. 2003). The value of FN in the urine and blood of UC patients still remains controversial. Possible reasons include the different methods used as well as the fact, that different FN types were measured including probably also abnormal forms, as demonstrated in other cell systems (Hegele et al. 2003). Therefore, though FN seems to be a promising marker, its value in the detection and follow-up of UC still needs prospective evaluation.

### Tenascin

Tn-C is a glycoprotein of the ECM, which is prominently expressed in developing tissues (Chiquet-Ehrisman and Chiquet, 2003). In adult tissues, expression of Tn-C is more restricted, but increases during processes associated with ECM remodeling such as inflammation and carcinogenesis (Chiquet-Ehrismann and Chiquet, 2003).

**Figure 1:**
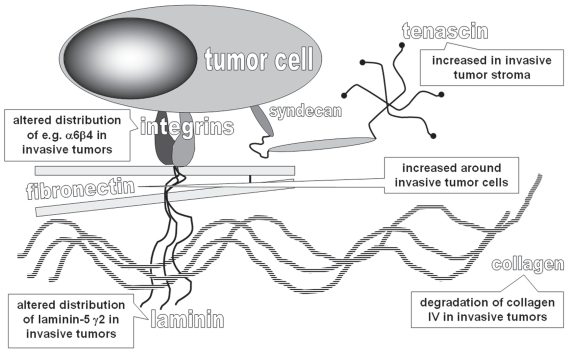
Scheme of extracellular matrix components potentially deregulated in invasive urothelial carcinoma of the bladder.

Tn-C is composed of six identical subunits, which are disulfid-linked, forming a large six-armed molecule. Subunits are built of repeated domains including epithelial growth factor-like repeats, heptad repeats, fibronectin III (FNIII) repeats and C-terminal globular domains shared with fibrinogen. Alternative splicing of Tn-C results in many different forms containing variable numbers of additional FNIII repeats (Chiquet Ehrismann and Chiquet, 2003). Functionally, Tn-C is involved in cell adhesion, migration and growth through the interaction with FN-dependent cell adhesion (Huang et al. 2001). Tn-C binds to the FN and syndecan-4, which is necessary for the cells to fully spread on FN (Huang et al. 2001). By interfering with the binding to FN, Tn-C prevents the interaction of cells with FN in synergy with integrin α5β1 (Huang et al. 2001; Chiquet-Ehrisman and Chiquet, 2003) (Figure 1).

In malignant tumors, the role of Tn-C is still under investigation and observations from studies on different tumor types remain conflicting, most likely as a result of the different Tn-C functions depending on different splicing variants and complex interactions (Brunner et al. 2004; Berndt et al. 2006). Tn-C expression has been reported at the invasive border of malignant tumors and was suggested to be of prognostic significance in several tumor types (Aishima et al. 2003; Wiksten et al. 2003). In the normal bladder, Tn-C was found in the superficial urothelial cells and the basal membrane (Wilson et al. 1994; Booth et al. 2002). Weak staining was also observed in the lamina propria and around vessels (Deen et al. 1994; Booth et al. 2002). UCs show an increase of Tn-C expression around invasive tumor cell nests as well as in the papillary cores and increased staining has been linked to tumor grade and stage (Tiita et al. 1993; Booth et al. 2002). So far, little data are available on the prognostic significance of Tn-C in UC.

In a retrospective study, we analyzed 106 UCs and showed that the staining performance for Tn-C of the tumor cells and stroma had a prognostic significance, depending on the specific microarchitectural localization and the pattern of Tn-C expression (Brunner et al. 2004). A diffuse Tn-C staining in the stroma of invasive tumors was associated with a significantly worse prognosis (Brunner et al. 2004). Beside stromal Tn-C expression, cytoplasmatic staining in superficial and invasive tumor cells was observed in this study (Brunner et al. 2004). The main source of Tn-C are stromal fibroblasts, though it has been shown that tumor cells are able to synthesize large amounts of Tn-C (Booth et al. 2002). The value of cytoplasmatic Tn-C remains questionable. We observed a better overall survival for patients with cytoplasmatic staining and a negative correlation between stromal and cytoplasmatic Tn-C (Brunner et al. 2004). We therefore suggest that the differences between stromal and cytoplasmatic Tn-C expression are probably due to different splicing variants of Tn-C, generating functional diversity with different effects on cell proliferation and migration. In breast cancer, Adams et al. found that different isoforms of Tn-C are expressed and that two isoforms (Tn16 and Tn14/16), exclusively synthesized in the stromal compartment, may be helpful in predicting invasion (Adams et al. 2001). Berndt at al. evaluated 34 UCs and found that, depending on the tumor type, invasive behavior and vascularization, different Tn-C splicing variants were expressed (Berndt et al. 2006). They suggested that this could be helpful in the assessment of invasion and could serve as target for antibody-mediated therapy (Berndt et al. 2006). In addition, they reported on specific splicing variants of Tn-C that were associated with newly formed vessels, further supporting the hypothesis that Tn-C plays a role in angiogenesis (Berndt et al. 2006). In fact, *in vitro* studies showed that endothelial cells spread on Tn-C via binding to integrins α2β1 and αVβ3 (Sriramarao et al. 1993). Tn-C expression in UC with particular emphasis on pattern and distribution may add prognostic information, though its role in tumorigenesis and progression of bladder cancer still requires further investigation.

### Other ECM molecules

A variety of other ECM molecules have been described in the human bladder, though data on their pathogenetic role and prognostic value in UC are scarce. These include vitronectin, elastin, and proteases other than matrix metalloproteinases, such as tetranectin (Wilson et al. 1996; Brunner et al. 2007).

One glycoprotein that has evoked increasing interest in UC of the bladder is thrombospondin 1 (TSP1) (Ioachim et al. 2006). TSP1 is a glycoprotein of the ECM known to be a potent inhibitor of angiogenesis (Ioachim et al. 2006). Immunohistochemical expression of TSP1 has been evaluated in UC of the bladder and was reported to predict recurrences and overall survival (Grossfeld et al. 1997; Goddard et al. 2002). Grossfeld et al. reported that low TSP1 was associated with high microvessel density, shorter recurrence-free survival and worse overall survival in a study including 163 UC patients (Grossfeld et al. 1997). They further found that the anti-angiogenic effect may be at least partly regulated by p53 (Grossfeld et al. 1997). Indeed, tumors with p53 accumulation were more likely to have reduced levels of TSP1 (Grossfeld et al. 1997). Goddard et al. found that loss of TSP1 was an independent prognostic factor indicating a worse overall survival (Goddard et al. 2002). *In vitro* studies have shown that TSP1 inhibits angiogenesis induced by a bladder cancer cell line and that TSP1, together with Tn-C, modulates sprouting of endothelial cells (Canfield et al. 1995; Campbell et al. 1998). Tn-C induces a sprouting phenotype inhibited by TSP1. Evaluation of FN, Tn-C and TSP1 in UC of the bladder showed a correlative expression, suggesting probable retained interactions between these ECM molecules during tissue remodeling (Ioachim et al. 2006). In contrast to previous results, Ioachim et al. reported a positive correlation of TSP1 with microvessel density and vascular endothelial growth factor (Ioachim et al. 2006). TSP1 may have inhibitory as well as enhancing effects on angiogenesis (Canfield et al. 1995). These effects, similarly to other ECM molecules, may largely depend on the composition of the surrounding matrix reflected by the association of TSP1 expression with FN and Tn-C.

### Integrins

Integrins are adhesion molecules that play a crucial role in the interaction between epithelial cells and ECM components as well as between different ECM proteins (Syrigos et al. 1999). De-arrangement, loss and shift of integrin expression are known to play an important role in the development of invasion of malignant tumors including UC of the bladder (Bosman and Stamenkovic, 2003; Syrigos et al. 1999). The most important ECM molecules, already discussed in this review, and their binding integrins including the proposed involvement in UC are summarized in Table 2. Integrins are transmembrane glycoproteins composed of non-covalently associated α/β heterodimers, made up of 15α and 9β chains (Bosman and Stamenkovic, 2003). Combinations of chains allow the formation of a wide range of different integrin molecules (Bosman and Stamenkovic, 2003). Integrins are found in all cells and are necessary for tissue structure maintenance (Bosman and Stamenkovic, 2003). They mainly function as receptors for ECM proteins, including laminin, collagen VII, FN, Tn-C, vitronectin and TSP1 (Syrigos et al. 1999). Some integrins bind only one specific ligand, while others have a variety of possible ligands (Syrigos et al. 1999). Differences in binding specificity may depend on the cell type by which the integrin is expressed. The normal bladder urothelium expresses α3, αV, β1 and β4, all being important for impermeability maintaining of the bladder wall (Wilson et al. 1996; Syrigos et al. 1999). α3-integrin has been suggested to be involved in the modulation of the expression of other integrin receptors in bladder cancer (Litynska et al. 2002).

De-arrangement of α6β4-integrin in bladder cancer was shown to be associated with invasive behavior, since its loss impairs tumor cell binding to the collagen VII basal membrane component (Liebert et al. 1994). It has been suggested that this is an early event in UC. Indeed, expression of integrins in UC becomes aberrant and deregulated with increasing grade and stage (Syrigos et al. 1999). Reduction of expression of integrin β4 has been found to correlate with an increased intraepithelial spread of tumors cells on laminin (Harabayashi et al. 1999). β1-integrin was reported to be critically involved in adhesion, extravasation and migration of an invasive bladder cancer cell line (Heyder et al. 2005).

Integrins are known to interact with ECM molecules such as FN and Tn-C (Syrigos et al. 1999). The function of FN is mediated by α5β1-integrin and syndecan-4, acting simultaneously (Huang et al. 2001). Tn-C interferes with syndecan-4 and prevents the FN-dependent adhesion and thus enhances proliferation. This finding as well as the increased expression of Tn-C and FN in the ECM of UC, suggests that the interaction between these two molecules is important for tissue remodeling in UC (Huang et al. 2001). Finally, integrins are involved in the sprouting of endothelial cells on Tn-C, suggesting that they are possible co-factors contributing to the angiogenic function of Tn-C, though so far studies in UC have not been performed (Sriramarao et al. 1993). All these findings point to the importance of an integrative approach for the elucidation of the role of ECM molecule expression in UC, considering also the impact of their associated receptor molecules.

### Perspectives

In UC of the bladder the most important prognostic factors still are based on morphology, including tumor size, multiplicity, associated carcinoma in situ, grade and stage (Rodriguez-Alonso et al. 2002). New prognostic factors including also ECM molecules could be of importance in estimating the risk of patients for relapse and muscle-invasive disease. In addition, the ECM in UC may be a potential therapeutic target for treatment in UC. Indeed, targeting the ECM, one can expect to bypass the treatment-resistance mechanisms of tumor cells. However, genetic changes have also been identified in the stroma surrounding tumor cells in mammary cancer and colonic polyps, and tumor cells can develop genetic alterations resulting in reduced stromal dependence (De Wever et al. 2003). Further investigations to better understand the stromal contribution on growth promotion, de-differentiation and invasion of urothelial carcinoma cells will be necessary to detailedly elucidate the complex role of ECM in UC.

### Summary

To summarize, ECM molecules in UC of the bladder seem to play an important role in the development of invasion, progression and metastasis. The mechanisms involved are complex, depending on the interaction of ECM proteins with each other as well as with cancer cells. Further studies, especially on FN, Tn-C and TSP1 and their interactions, mediated by integrins, are necessary to elucidate the different roles of these ECM components during tumor invasion and angiogenesis, providing new insights in carcinogenesis and probably leading to new therapeutic approaches.

## Figures and Tables

**Table 1:** Distribution of structural extracellular matrix (ECM) components, function and prognostic value in urothelial carcinoma of the bladder.

	Normal localization	Localization/function in malignant tumors	Prognostic value	References
Laminin	Basal membrane	Laminin-5 γ2 chain: altered distribution out of basal membrane, loss of cellular retention	Altered distribution of laminin-5 γ2-chain associated with worse overall survival, higher risk of recurrence and progression; independent prognostic factor in bladder cancer treated with TUR	Schapers et al. 1990; Hindermann et al. 2003; Kiyoshima et al. 2005; Abou Fahra et al. 1993
Collagen	Basal membrane (IV, VII) ECM (I, III)	Loss of collagen IV in basal membranes, de- arrangement of collagen VII	Loss of collagen IV associated with invasive behavior and worse overall survival	Schapers et al. 1990; Daher et al. 1987; Alampi et al. 1989
FN	Lamina propria, attenuated suburothelial	Increased in lamina propria, co-expression with Tn-C, increased in urine	Increased expression associated with stage, proliferation, Tn-C, laminin and collagen IV expression, no prognostic value; increased in urine: early detection of tumor; decrease in urine: response to Bacillus Calmette-Guérin-therapy	Ioachim et al. 2001; Hegele et al. 2003; Menendez et al. 2003; Malmstrom et al. 1993
Tn-C	ECM	Different splicing variants, increased around invasive tumor cell nests loss of cellular retention	Increased stromal expression associated with worse overall survival; tumor cell expression associated with improved overall survival	Brunner et al. 2001
TSP1	ECM, tumor cells, around vessels, at stromal-epithelial junctions	Decreased perivascular and at stromal-epithelial junction	Decreased expression associated with high rate of recurrence and worse overall survival	Goddard et al. 2002; Grossfeld et al. 1997; Ioachim et al. 2006

**Abbreviations:** FN: fibronectin; Tn-C: tenascin-C; TSP1: thrombospondin 1; TUR: transurethral resection.

**Table 2:** Extracellular matrix (ECM) molecules and associated integrins as well as mediated function involved in tumor development.

	Integrins	Functional importance in tumors	References
Laminin	α1–7β1, α6β4	Laminin-5 γ2 chain: part of anchoring filaments, interaction between ECM and tumor cells, cellular differentiation, migration	Hindermann et al. 2003; Kiyoshima et al. 2005; Wehrle-Haller et al. 2003
Collagen IV	α1–2β1, α10–11β1	Loss, fragmentation in invasive tumors	Conn et al. 1987; Wehrle-Haller et al. 2003
Collagen VII	α6β4	De-arrangement probably involved in invasion	Liebert et al. 1994
FN	α3–7β1, α5β1, αIbβ3, α5β3, α4β7, αVβ6	Cellular adhesion, proliferation, differentiation	Chiquet-Ehrismann et al. 1988; Wehrle-Haller et al. 2003
Tn-C	α2β1, αVβ3, α9β1	Endothelial cell attachment and spreading, modulation of cell proliferation and motility	Sriramarao et al. 1993; Wehrle-Haller et al. 2003; Gulubova et al. 2006
TSP1	α5β3	Anti-angiogenesis interactions with Tn-C in endothelial cell sprouting	Wehrle-Haller et al. 2003; Grossfeld et al. 1997; Canfield et al. 1995

**Abbreviations:** FN: fibronectin; LN: laminin; Tn-C: tenascin-C; TSP1: thrombospondin 1.
